# History and structure of the closed pedigreed population of Icelandic Sheepdogs

**DOI:** 10.1186/1297-9686-41-39

**Published:** 2009-08-06

**Authors:** Pieter A Oliehoek, Piter Bijma, Arie van der Meijden

**Affiliations:** 1 Animal Breeding and Genomics Centre, Wageningen University, the Netherlands; 2 Faculty of Geography/Geosciences, Biogeography Department, Trier University, Germany

## Abstract

**Background:**

Dog breeds lose genetic diversity because of high selection pressure. Breeding policies aim to minimize kinship and therefore maintain genetic diversity. However, policies like mean kinship and optimal contributions, might be impractical. Cluster analysis of kinship can elucidate the population structure, since this method divides the population in clusters of related individuals. Kinship-based analyses have been carried out on the entire Icelandic Sheepdog population, a sheep-herding breed.

**Results:**

Analyses showed that despite increasing population size and deliberately transferring dogs, considerable genetic diversity has been lost. When cluster analysis was based on kinships calculated seven generation backwards, as performed in previous studies, results differ markedly from those based on calculations going back to the founder-population, and thus invalidate recommendations based on previous research. When calculated back to the founder-population, kinship-based clustering reveals the distribution of genetic diversity, similarly to strategies using mean kinship.

**Conclusion:**

Although the base population consisted of 36 Icelandic Sheepdog founders, the current diversity is equivalent to that of only 2.2 equally contributing founders with no loss of founder alleles in descendants. The maximum attainable diversity is 4.7, unlikely achievable in a non-supervised breeding population like the Icelandic Sheepdog. Cluster analysis of kinship coefficients can provide a supporting tool to assess the distribution of available genetic diversity for captive population management.

## Background

Closed populations with high levels of genetic drift suffer from reduction of genetic diversity. Genetic diversity is essential to maintain the adaptive potential of populations, and confers higher resistance to pathogens. In the end, reduction of genetic diversity causes higher levels of inbreeding, which can cause inbreeding depression as well as high incidences of particular heritable (often recessive) diseases. Managing genetic diversity within populations is necessary to avoid high incidences of deleterious alleles and to preserve adaptive potential.

In managed populations, such as domestic animals, genetic diversity can be maximised by selection according to optimal contributions, giving each reproductive animal a specific contribution for the next generations [1,2]. However, for many populations, this optimal approach cannot be applied as a breeding strategy, because there is not one single authority that can decide which animals to select for breeding. These populations can still increase their genetic diversity with sub-optimal solutions, which require an overview of the genetic diversity within these populations. Hence, individual breeders need insight in the population structure and in how genetic diversity can be maintained.

Ubbink *et al*. [3-5] have used cluster analysis of kinship coefficients to elucidate the relational structure of purebred dog populations, and to demonstrate correlation with a genetic disease present in these populations. Instead of 'looking at a large pile of pedigrees' or a table with mean kinships [6], they used hierarchical cluster analysis to visualise the hitherto unknown structure of pedigreed populations into separate highly related clusters ('family groups') that have a certain level of kinship (relationship) among each other.

A dog breed is an example of an 'unsupervised' closed population [7] in which mating is only allowed between registered dogs of the same breed. Purebred dogs are subject to strong selection to meet the breed standards. Dog breed populations can go through a permanent reduction of genetic diversity due to three factors: (1) only a small fraction of all pure-bred males and females actually reproduce [4]; (2) there is an unequal number of litters among reproductive males [8]; and (3) dog breeds are often fragmented [9]. This permanent reduction of genetic diversity (bottleneck) has resulted in a high incidence of specific genetic diseases in different breeds, and in some breeds most of the animals are affected or carriers [10]. It is now well recognised that genetic diseases are a major threat for purebred dog populations [11].

Icelandic Sheepdogs are bred in several European countries by many individual breeders. It is well known that the current population of Icelandic Sheepdogs descends almost entirely from only a few founders that were selected from remote areas in Iceland between 1955 and 1965.

In the work presented here, we investigate the amount of genetic diversity lost and the possibilities to maintain or increase genetic diversity within the Icelandic Sheepdog population considered as a typical closed dog population. Furthermore, cluster analysis is evaluated as a tool and for its potential to identify genetic diversity.

## Methods

### Data

We received pedigree data via ISIC [12] of the population of Icelandic Sheepdogs in the following countries: the Netherlands (725 records), Sweden (1367), Iceland (1654), Germany (153), Norway (774), Denmark (2241) and Finland (113). Pedigree data contained unique ID, father, mother, gender, date of birth, country of birth, and occasionally date of death. Only Iceland had data since 1955. In other countries, breeding started in 1975 or later and most of the data went up to 2002 and some only up to 1998. Except for a few dogs in France, these countries cover the entire Icelandic Sheepdog population. Animals without recorded parents were classified as either (1) 'original founders': animals without any relationship with other founders, documented as such by the kennel clubs, or (2) 'related animals with unknown parents': animals that descend from the 'original founders' or their progeny, but having unknown parentage. Furthermore, some individuals were registered in more than one country. The pedigree data were assembled into a single database table, and animals that were recorded twice were removed based on information on the country of birth. The problem of 'related animals with unknown parents' was solved by assembling all datasets with additional information on parentage from ISIC. After this process, only the original founders had unknown parents. The equivalent complete generations traced for each animal was computed as the sum of the proportion of ancestors known per generation [13]. Until 1998, pedigrees were complete for all countries. A general life expectancy was estimated separately for males and for females from the interval between date of birth of parents and progeny. If date of death was not recorded, it was estimated by life expectancy. All animals born between 1991 and 1998 were considered as the 'current-population'.

### Population diversity measures

Unless otherwise stated, inbreeding and kinship coefficients were calculated using the tabular method. Except for optimal contributions, which were calculated using Fortran, all measures were calculated using Visual Basic. Mean kinship was proposed by Ballou and Lacy [6] and is the mean of the kinship coefficients between that individual and all candidates, including the individual itself. Candidates are defined as reproductive individuals of the current population. The mean kinship (*mk*_*i*_) for individual *i *is calculated by Ballou and Lacy [6] as:

(1)

where *N *is the number of candidates and *f*_*ij *_is the kinship between individual *i *and individual *j*. The mean kinship of an animal is a measure of the relationship of that individual with a population; animals with a low mean kinship are more valuable for genetic diversity. Mean kinship depends on the population which means that the mean kinship of an animal might change over time when a population changes. In conservation genetics, mean kinship is an important tool to maintain genetic diversity [14].

The following population diversity measures were used:

Average inbreeding () is the average of the inbreeding coefficient of all candidates.  indicates the current risk of inbreeding depression in the current population.

Average mean kinship () is the average of mean kinships of all candidates within the population under study [6], and was calculated as:

(2)

Average *mean *kinship, which is predominantly used in conservation [2,6], differs from average *pairwise *kinship because  includes kinship of animals with themselves.

In this work, genetic diversity (*N*_*mk*_) is defined as the number of equally contributing founders with no random loss of founder alleles in descendants that would be expected to produce the same average mean kinship (and therefore genetic variation) as in the population under study. *N*_*mk *_is  expressed on the scale of founder genome equivalents [15,16] and is calculated by *N*_*mk *_= 1/2. A lower average mean kinship means a higher genetic diversity and thus a higher capacity to adapt as a population.

In this work, allelic diversity (*N*_*AD*_) is defined as half the number of distinct alleles that are still present in the population under study if all founder alleles were unique. The number of unique founder alleles that survive each year was determined by genedrop [17], which was repeated 10.000 times. *N*_*AD *_is also expressed in founder genome equivalents and can therefore be compared with *N*_*mk *_and *N*_*OC *_(see below). For example, if the frequencies of all alleles were equal, *N*_*AD *_would be equal to *N*_*mk*_. *N*_*AD *_monitors the loss of genetic diversity due to extinction of unique (founder-) alleles.

In this work, potential diversity (*N*_*OC*_) is defined as the maximum genetic diversity the population under study can achieve (expressed in founder genome equivalents). *N*_*OC *_is the genetic diversity obtained when average mean kinship is minimised using Optimal Contribution Selection. *N*_*OC *_is calculated as described in Oliehoek *et al*. [18]:

(3)

where **F **is a matrix of kinships between all individuals, including kinship of individuals with themselves, and **c**_*OC *_is a column vector of proportional contributions of individuals to the next generation, so that the sum of elements of **c**_*OC *_equals one and minimises **c**_*OC*_**'Fc**_*OC *_[19]. **c**_*OC *_is given by Eding *et al*. [20]:

(4)

where **1 **is a column vector of ones. **c**_*OC *_contains contributions of parents to next generations that would minimise  in next generations. However, **c**_*OC *_calculated from Equation 4 can contain negative contributions, which is impossible in practice. When negative contributions were obtained, the most negative contribution was set to zero and vector **c**_*OC *_was recalculated until all contributions were non-negative. *N*_*OC *_is the highest possible *N*_*mk *_and measures the diversity that could be obtained in next generations. *N*_*OC *_will always be equal or higher than *N*_*mk *_and equal or lower than *N*_*AD*_. *N*_*OC *_is relevant in the case of closed populations, since the population can never reach a diversity higher than *N*_*OC*_. Therefore, it monitors the unrestorable loss of genetic diversity.

### Diversity and Population History

For each year a 'current population' was defined as all the animals expected to be alive and the following population-parameters were determined: the current population size; the number of progeny born during that year; the number of founder introductions; and the following diversity measures: , , *N*_*mk*_, *N*_*OC*_, *N*_*AD *_(as described above).

### Cluster-analysis

Cluster-analysis was performed twice on the current population. (1) The first analysis was based on kinship calculated using the tabular method starting with the founders and then UPGMA was applied for clustering all animals [21]. To determine the most appropriate number of clusters, *R*^2^, the cubic clustering criteria and pseudo-*F *statistic were all examined (SAS Institute, release 9.1, Cary, NC, USA). These clusters are displayed in a dendrogram, which is referred to as the all-gen-tree. (2) The second cluster-analysis was performed as described by Ubbink *et al*. [4]. Kinships between all animals were calculated by the path method [22] until seven generations backwards (instead of the tabular method that includes all generations). Note that if the path method included all the generations, results would be equal to the tabular method. Then, all the animals were clustered using UPGMA. Subsequently all the clusters having an average mean kinship greater or equal to 0.0625 were defined as the final clusters and displayed in a dendrogram. This kinship value of 0.0625 that delimits clusters corresponds with kinship between second degree cousins and was used by Ubbink *et al*. [4]. This dendrogram is referred to as the 7-gen-tree.

## Results and discussion

### Data and current population

Of the 4680 dogs in the data, 36 did not have any parents registered and were recognised as founders by the breeding organisations. All other dogs in the pedigree file descended from these 36 founders. Most founders lived in Iceland and were registered there, except for four animals that lived in Germany.

The current population contained 2554 dogs and represented 512 unique parent combinations. For dogs in the current population, the most 'distant' founders appeared in their pedigree 10 to 20 generations back (nine to 19 ancestors between the current animal and the founder). The equivalent complete generations [13] traced was 9.1.

All the animals of the current population can only carry alleles from the 36 founders. In the Icelandic Sheepdog, just three of the 36 founders contributed more than 80% of the alleles of the current population (results not shown). In other words, in about 80% of cases, the pedigree of every animal in the current population will end with one of these three over-represented founders.

### Population history

**Figure 1:**
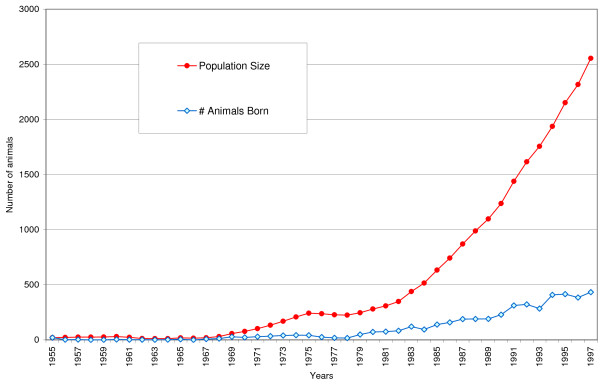
**History of population-size**. Population Size is the number of animals that were (likely to become) reproductive; # Animals Born indicates the number of puppies that were born during that specific year

**Figure 2:**
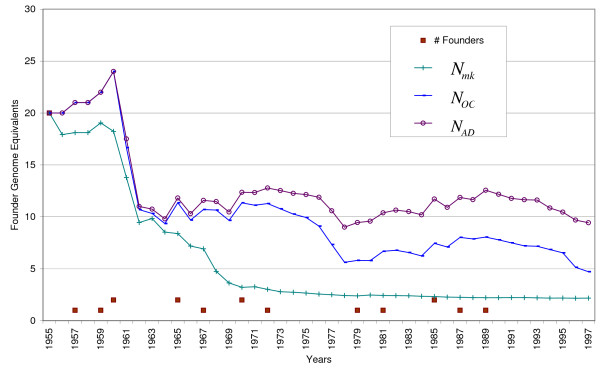
**History of diversity in founder genome equivalents**. # Founders is the number of founders introduced during that specific year; after 1991 no new founder was introduced; *N*_*mk *_is the average mean kinship in founder genome equivalents; *N*_*OC *_is the minimum possible kinship in founder genome equivalents; *N*_*AD *_is half the number of distinct alleles if founders had unique alleles (scale of founder genome equivalents)

Figure 1 shows the population size and the number of animals born. The population size hardly grew until 1967, and then reached 250 animals. Until 1980, most Icelandic Sheepdogs lived in Iceland but after, their number increased in other countries as well. Figure 2 shows the number of founder introductions, together with genetic diversity (*N*_*mk*_), potential diversity (*N*_*OC*_), and the allelic diversity (*N*_*AD*_). In 1955, the first 20 founders were selected for breeding. These animals were chosen from remote areas in Iceland.

Figure 2 has eight points of interest. (1) When 20 founders were selected this resulted in equal, *N*_*mk*_, *N*_*OC *_and *N*_*AD *_(all equal to 20). (2) *N*_*mk *_has decreased since 1955, despite 10 founder introductions up till 1973 and six more after 1979. Each newly introduced founder can potentially increase genetic diversity but clearly in this case, founder introductions have not increased *N*_*mk*_. (3) However, each founder introduction increases *N*_*OC *_and *N*_*AD *_by one. (4) Between 1960 and 1964, *N*_*OC *_and *N*_*AD *_have decreased from 24 to less than 10. This remarkable drop is explained by the fact that most of the 20 founders that were introduced in 1955 only produced one offspring and then died during this period. (5) *N*_*mk *_has strongly decreased from 6.9 in 1967 to 3.2 in 1970. This is contemporaneous with the start of the first population size growth. *N*_*OC *_and *N*_*AD *_did not decrease as much during that period. Therefore, the decrease of *N*_*mk *_is caused by unequal allele frequencies and not by extinction or mixing of unique alleles with over-represented alleles. The strong decrease of *N*_*mk *_is due to a disproportional contribution of a small number of individuals to the future generation. (6) Unequal representation of founder animals in offspring is also responsible for the decrease of *N*_*mk *_during the first years. (7) The distance between *N*_*OC *_and *N*_*AD *_has increased ever since 1963 and reached 5.2 in 1997, which means that it became increasingly difficult to equalise allele frequencies. In other words, 5.2 founder genome equivalents were lost because of unique alleles mixing with over-represented alleles within individuals. Optimal Contribution Selection cannot restore this loss. (8) The difference between *N*_*mk *_and *N*_*OC *_shows that this population has the potential to increase genetic diversity.

**Figure 3:**
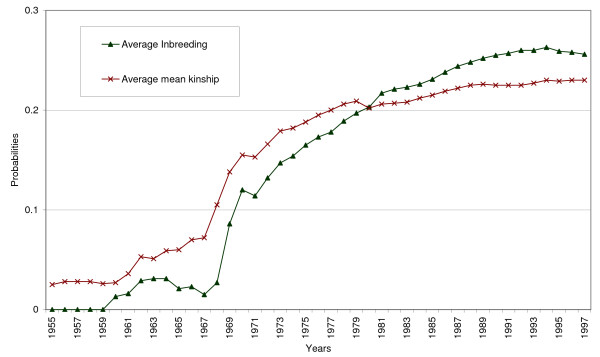
**History of inbreeding and kinship of the current population**.

Figure 3 shows , which is the average mean kinship expressed in probabilities instead of founder genome equivalents (*N*_*mk*_), in order to compare  with average inbreeding (). Inbreeding starts at 0 and is initially lower than kinship, which is expected because kinship includes kinship of individuals with itself. Later inbreeding increases at a higher rate than kinship, and the average inbreeding becomes higher than the average mean kinship (in percentage), from 1980 till 1997. This phenomenon can be attributed to geographic subdivision within the population. Breeding occurs mainly between dogs within a given country, and the dogs are more related to each other.

### Cluster Analysis Methods Compared

**Figure 4:**
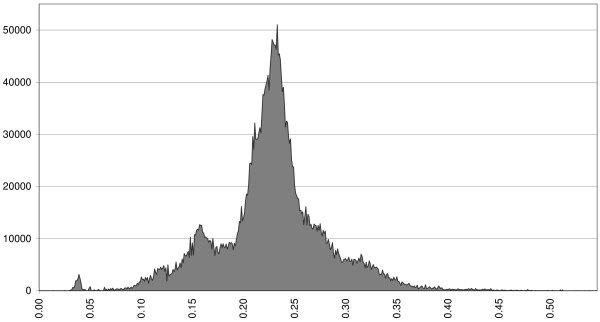
**Histogram of pairwise kinship values among all dogs of the current population**. The histogram shows pairwise kinships ranging between 0 and 0.55 with a class interval of 0.001; the area under the curve equals the total number of observed pairwise kinship among all 2554 dogs of the current population

**Figure 5:**
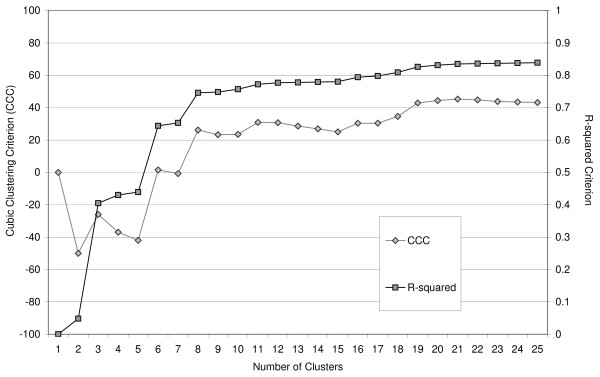
**Cluster criteria**. The cubic clustering criterion (CCC) and the *R-*squared per number of clusters (1 to 25).

Figure 4 is a histogram of all pairwise kinship values calculated by using all generations among the 2554 dogs of the current population. This histogram is multi-modal, which indicates the existence of clusters. Figure 5 gives the cubic clustering criterion and the *R*^2^-values for different numbers of clusters (1 to 25). At cluster numbers of 3, 5 and 8, *R*^2 ^shows a jump. Up to eight cluster numbers, cubic clustering criterion is around zero or less. However, when the number of clusters equals 8, it increases to 26.2, which means that the *R*^2 ^is larger than can be expected from a normal distribution. The pseudo-*F *statistic was highest at a cluster number of 8 (1066). Eight clusters were selected based on these three criteria.

**Figure 6:**
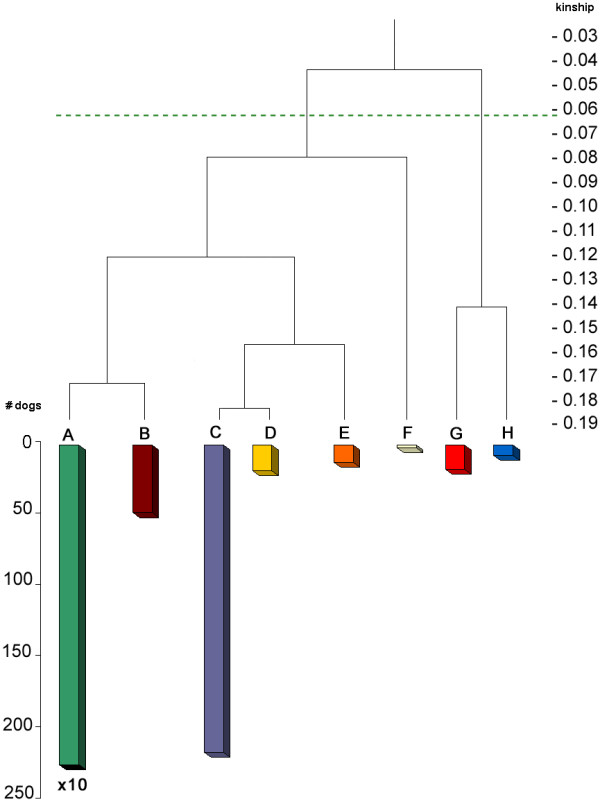
**Cluster analysis of current population (all-gen-tree)**. Results of clustering based on kinship coefficients calculated using the tabular method (all generations included); the legend with codes per cluster was added in order to compare this dendrogram to that in Figure 7; the length per cluster corresponds with the number of (reproductive) individuals, except for cluster A, which is 10 times the size depicted, representing 2236 animals; he line at the 0.0625 kinship level, corresponds with the 'cut-off level' of the cluster analysis of Figure 7

**Figure 7:**
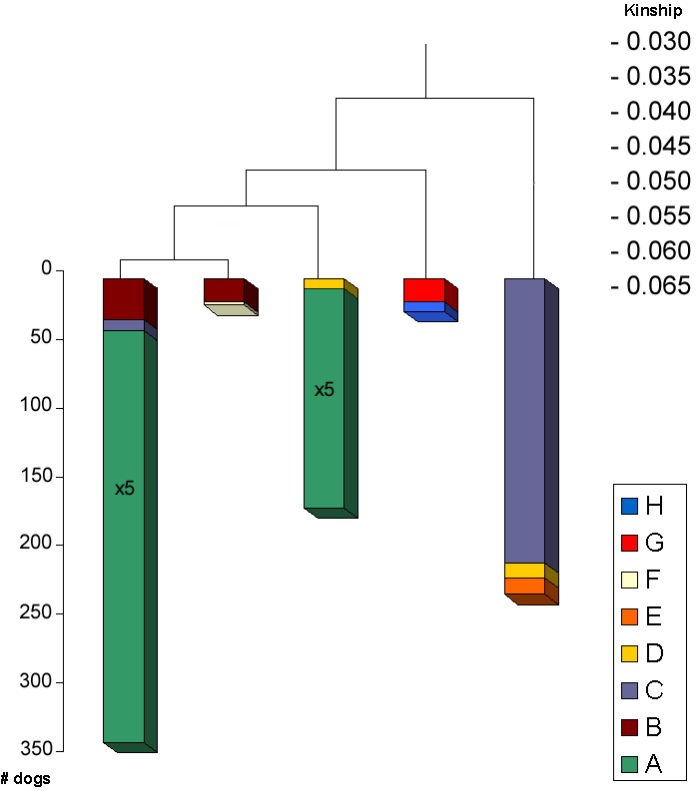
**Cluster analysis of current population based on 7 generations (7-gen-tree)**. Results of clustering based on kinship coefficients calculated by the path method for seven generations backwards; the legend represents the clusters as demonstrated in Figure 6; the length per cluster corresponds with the number of individuals, except for the first and the third cluster from the left: the length of the 'green' A fraction corresponds to five times the actual size

Figure 6 shows the all-gen-tree, which is the dendrogram from the cluster analysis of the current population based on kinship coefficients calculated by the tabular method starting with the founders (all generations) having eight clusters: A to H. Figure 7 shows the 7-gen-tree, which is the dendrogram from the cluster analysis of the current population based on kinship coefficients calculated by the path method from the current population back to seven generations. The all-gen-tree clusters (A to H) are inserted for each dog to each cluster in the 7-gen-tree. Each cluster represents a number of animals that are highly related to each other. Branches indicate the kinship among the clusters. The 7-gen-tree differs substantially from the all-gen-tree. The all-gen-tree consists of one large cluster A, representing 2236 animals and a few smaller clusters (representing altogether 318 animals). However, in the 7-gen-tree, this cluster A is split at a much lower kinship-level i.e. 0.055. The smaller clusters of the all-gen-tree, redistribute and sometimes split themselves in the 7-gen-tree.

Ubbink *et al*. [4] have shown that, in their population, the inclusion of five, six or seven generations yielded virtually identical and reproducible results. Hence, Ubbink *et al*. [4] have suggested that it is sufficient to calculate kinship seven generations backwards. Based on the substantial difference between the 7-gen-tree and the all-gen-tree in our study, we conclude that this assumption does not hold for the present population. This difference can be explained by the presence of common ancestors that are undetected at five, six or seven generations. An example of such undetected ancestors is given by the strong influence of the three predominant founders. At least 80% of the alleles of the current population descend from these three founders. While these founders dominate the pedigree many generations back, they remain undetected at five, six or seven generations. These three founders, possibly together with other frequently used ancestors, cause the difference between the 7-gen-tree and the all-gen-tree. The cluster analysis based on all generations is therefore a better representation of real kinship.

### Diversity per cluster

**Figure 8:**
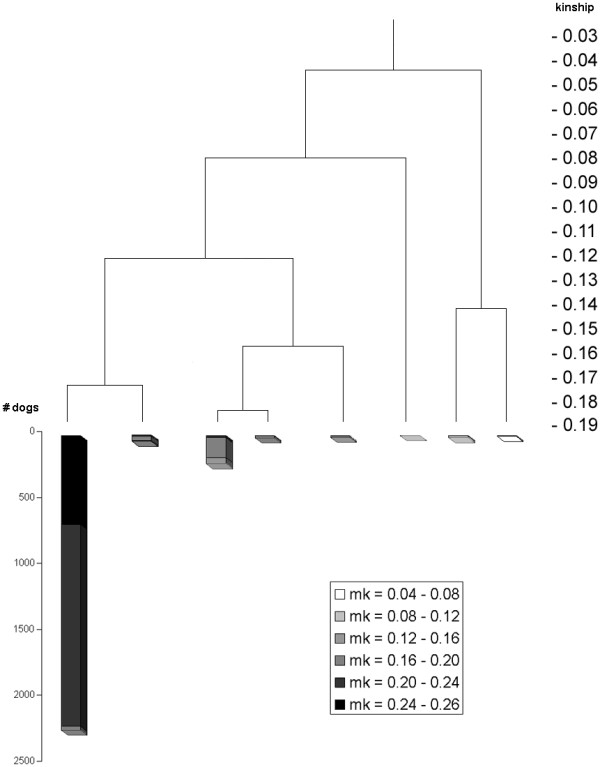
**Dendrogram based on all generations showing mean kinship**. Results of clustering based on kinship coefficients calculated by the tabular method (all generations included) of all reproductive Icelandic Sheepdogs; mean kinship per animals was implemented; grey-scales indicate the mean kinship for each animal; higher mean kinships show darker and therefore less important genetically

Table 1 gives the diversity measures: , , *N*_*mk*_, *N*_*OC*_, *N*_*AD *_for each of the eight clusters treating each cluster as a separate 'population'. Note that mean kinship depends on this cluster. In Table 1 mean kinship is calculated within each cluster; thus mean kinship calculated per cluster differs from mean kinship calculated for the current population as shown in Figure 8 (see below). The  of cluster A is only a little higher than the  of the population. Since cluster A contains 85% of the population, it largely determines the  of the population. Table 1 shows that while average inbreeding differs per cluster, the average mean kinship is roughly the same for most clusters; *N*_*mk *_is between 1.7 and 2.0. Only the cluster F, which contains only two animals, has an *N*_*mk *_of 1.2. This is because kinship of an animal with itself has a higher effect on the total kinship in small populations. No single cluster can contain all the potential diversity. Moreover, within each cluster, the potential diversity *N*_*OC *_is hardly higher than *N*_*mk*_, whereas for the population as a whole *N*_*OC *_*is *more than double *N*_*mk *_(4.7 vs. 2.2). This indicates that an increase of genetic diversity in the current population can be achieved by optimisation between clusters but not by breeding within clusters. Each cluster could potentially contribute to genetic diversity. The small difference of *N*_*mk *_and *N*_*OC *_within clusters also indicates that all dogs within the cluster are strongly related to each other.

**Table 1:** Diversity measures within each cluster of dendrogram 4

Cluster:	**A**	**B**	**C**	**D**	**E**	**F**	**G**	**H**	All*^1^
#Animals	2236	47	215	18	12	2	17	7	2554
	.27	.16	.21	.12	.11	.02	0	0	.26
	.25	.26	.28	.30	.27	.39	.25	.29	.23
*N*_*mk*_	2.0	1.9	1.8	1.7	1.8	1.3	2.0	1.8	2.2
*N*_*OC*_	2.4	2.1	2.2	1.7	1.9	1.3	2.0	1.8	4.7
*N*_*AD*_	5.6	3.4	3.5	2.6	2.6	1.5	2.4	2.0	9.4
	
Relative size	87.5%	1.8%	8.4%	0.7%	0.5%	0.1%	0.7%	0.3%	100%
Contribution*^2^	16%	7%	9%	0%	17%	16%	12%	23%	100%

is average inbreeding (in probabilities);  is the average mean kinship *within *this cluster (expressed in probabilities); *N*_*mk *_is the average mean kinship *within *this cluster (expressed in founder genome equivalents); *N*_*OC *_is the minimum possible kinship *within *this cluster (expressed in founder genome equivalents); *N*_*AD *_is half the number of distinct alleles if founders had unique alleles *within *this cluster (expressed in founder genome equivalents)

*1 show values per diversity measure for the entire population

*2 Contribution is the sum of contributions that specific animals within their cluster would receive after application of optimal contributions over the entire population

### Ideal conservation of the Icelandic Sheepdog

Although genetic diversity (*N*_*mk*_) of the current population of the Icelandic Sheepdog was only 2.2, the potential diversity (*N*_*OC*_) was 4.7. In other words, *N*_*mk *_could be increased from 2.2 to *N*_*mk *_= 4.7. However, this value can be achieved within a few generations only if specific animals are used for breeding according to their specific optimal contribution (as in vector: **c**_*OC*_) as calculated for each of the 2554 animals. Table 1 shows for each cluster in the all-gen-tree: a) the relative size of each cluster toward the current population in percentage and b) the optimal contributions per individual summed per cluster. Table 1 shows that animals within the small clusters E to H, would have to contribute for 12% up to 23% per cluster, while their cluster sizes are smaller than 1% of the total population size. The optimal contribution per animal ranged from zero to 8% (of a total of 100%). In the ideal situation, 2410 animals of the 2554 would not contribute, while 50 animals would contribute for 80% in future generations. This optimal breeding scheme would require a complete control over the population. This scheme based on optimal contributions will most probably not be applied in multi-breeder ('unsupervised') populations like dog breeds because many breeders would not be allowed to breed at all.

### Cluster analysis combined with country of birth

**Figure 9:**
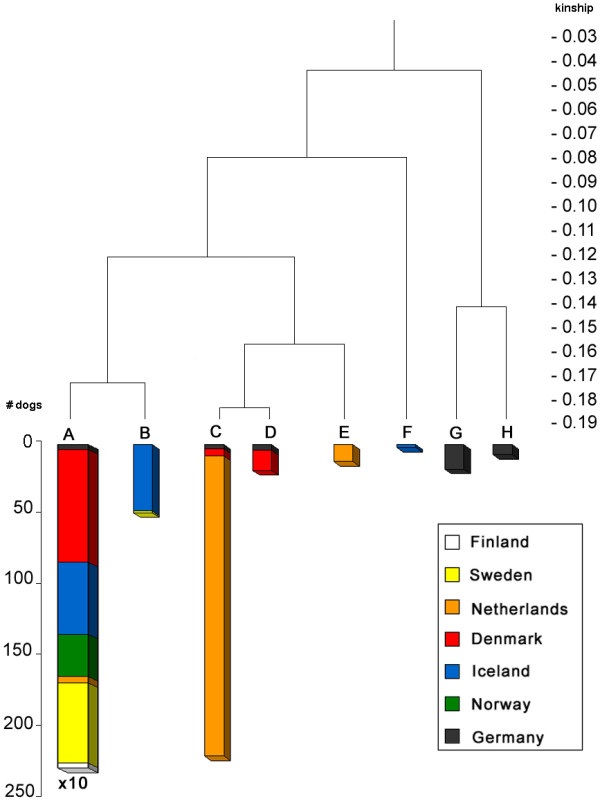
**Dendrogram based on all generations showing country of birth**. Results of clustering based on kinship coefficients calculated by the tabular method (all generations included) of all reproductive Icelandic Sheepdogs; colours indicate the fractions of each family group born in a particular country; the length per cluster and each country per cluster correspond with the number of individuals, except for cluster A that represented 2236 animals and each country within cluster A was scaled down by a factor 10

Figure 9 shows the all-gen-tree (as in Figure 6), including the country of birth for each dog in each cluster. It illustrates the geographic distribution of kinship clusters of the current population. One large cluster (cluster A) contains almost every dog of Scandinavia and contains 85% of the total population size. It includes the entire Norwegian and Finnish populations and almost every animal born in Sweden or Denmark, and a large part of the population of Iceland. Cluster B contains the rest of the Icelandic population, except for the distant cluster F that consists of two full-sibs born in Iceland. The related clusters C and E mainly contain the Dutch population. Most German Icelandic Sheepdogs are found in the most distant clusters G and H. German and Dutch populations are less related to Scandinavian populations mainly because the five founders that were introduced between 1970 and 1990 in Germany were unrelated to other founders. However, those founders were not recognised by the Iceland kennel club as being true Icelandic Sheepdogs and thus, were not often used outside Germany.

The reason why a single large Scandinavian cluster exists is not only due to the founder-effect. Many sheepdog imports from Iceland were carried out to increase diversity ("new blood") within each country. Breeders often think that within one country dogs are more related to each other and belong to the same cluster and they are often unaware that dogs from other countries might also belong to the same cluster. Since importing a dog is a large investment, breeders always selected the 'best dogs' from Iceland. Without knowing, Scandinavian mainland-countries imported highly related dogs time and again. This close relationship was not obvious on the standard pedigree forms given out by studbooks, because they indicate only three or at the most five generations. This lack of knowledge about true kinship among animals explains the occurrence of one large highly related cluster. Undetected relatedness is also the cause for the significant difference between cluster-analysis based on seven or on all generations (Figure 1 and 2). For several generations, related animals appear unrelated because pedigrees only go back three to five generations. Founder and other ancestors from previous generations might contribute significantly to kinship but are not detected at this level.

### Mean kinship and cluster analysis

Mean kinship per animal was calculated for the current population. Figure 8 shows the all-gen-tree dendrogram (as in Figures 6 and 7) with mean kinships per animal displayed in each cluster. Note that mean kinships differ from those in Table 1 where mean kinship was calculated *within *each cluster. The distance of each cluster to cluster A decreases mean kinship of animals of that cluster. This means that a conservation strategy based on selecting animals from distant clusters would give similar results than that based on selecting animals with a low mean kinship. While selection by optimal contributions is not possible within a multi-breeder population, cluster analysis could help in increasing genetic diversity. Cluster analysis can provide insight in the population structure for individual breeders, which helps to persuade them to select dogs from distant clusters.

In the populations of other breeds studied by Ubbink *et al*. [3,4], specific genetic diseases could be linked with some specific clusters and breeders were advised not to use any dogs from a cluster associated with the disease. Table 1 and Figure 8 show that populations might lose more diversity than breeders would expect when such a decision is based on a cluster analysis performed only with seven generations. This emphasizes the importance of including all generations in kinship calculation, or at least as many generations as possible.

### Genetic diversity compared with other populations

Lacy [16] has recommended to maintain *N*_*mk *_= 20 to guaranty adequate genetic variability. *N*_*mk *_of the Icelandic Sheepdog was only 2.2. Leroy *et al*. [23] have found a higher value (*N*_*mk *_= 5.2 to 25) for nine French dog breeds. However, these results are difficult to compare since the correction for 'related animals with unknown parents' was not implemented because they were treated as founders [24]. Głażewska [25] have reported a founder genome equivalent of 1.3 in Polish hound, which is comparable with the *N*_*mk *_of 1.3 and concludes that Polish hound has a dramatic low level of genetic variability. Overall, it is surprising that, at the time of our study, the Icelandic Sheepdog did not show any genetic disease considering its level of inbreeding. Fortunately, the population size is still increasing, which usually lowers genetic drift.

## Conclusion

The overall picture of the Icelandic Sheepdog breed is as follows. The Icelandic Sheepdog breed was built from founders, located on remote areas of Iceland between 1955 and 1970. A good part of the diversity was already lost during the first years of the development of the breed. Figure 2 shows that about 16 of the original 26 founder genomes were lost by 1966. In a recent study [26] of a subset of 133 dogs born in Iceland, the average inbreeding coefficient was 0.21, which is in agreement with the average inbreeding found in clusters A, B and C (Table 1). Breeding preferentially a few (and often related) animals, led to further reduction of genetic diversity. Thus, the potential diversity of Icelandic Sheepdogs, which was mainly present in animals from Iceland was not disseminated and in fact, decreased even within Iceland. In 1998, the *N*_*OC *_was only 4.7 and genetic diversity was less than half of that and equalled *N*_*mk *_= 2.2. Thus, in other words: the current population had a genetic diversity equal to 2.2 equally contributing founders with no random loss of founder alleles in descendants. An increase of genetic diversity to *N*_*mk *_= 4.7 is not possible within a few generations in a multi-breeder population like the Icelandic Sheepdog.

Breeding with animals having a low mean kinship is an important conservation method [14]. Cluster analysis is consonant with mean kinship: distant clusters contain animals with a low mean kinship and potential diversity within clusters is hardly higher than genetic diversity (Table 1), while within the current population as a whole, potential diversity is almost twice the current diversity. Cluster analysis of kinship coefficient based on all generations reveals the population structure and provides better insight on where to find genetic diversity. The all-gen-tree of Figure 9 shows that the genetically important animals are mainly in Iceland, Holland and Germany. Therefore, cluster analysis is suitable especially for exchanging information on genetic diversity in small closed pedigreed multi-breeder populations.

Although conservation of genetic diversity by means of optimal contribution selection is unlikely to happen within a multi-breeder population, preservation of potential diversity may be the second best option, when few animals are involved. In the Icelandic Sheepdog, optimal contributions show that the number of individuals with the highest potential genetic diversity equals about 50. It remains to be seen whether it is possible to convince some breeders to use those animals for breeding or for cryo-conservation of semen and oocytes.

This research underlines that dog breeds suffer from genetic drift continuously. Often dog breeding is only authorized with animals meeting specific criteria. These selection criteria, like show-qualifications and health status reports, often strongly limit the number of animals used in breeding. Moreover, certain specific animals are genetically important (see also Table 1), but in practice, these animals are often not used at all because they do not meet the previously mentioned selection criteria. Therefore, selection criteria might unintentionally accelerate loss of genetic and/or potential diversity, which is harmful for populations as a whole.

## Competing interests

The authors declare that they have no competing interests.

## Authors' contributions

PA conceived the study and carried out the research, PB participated in its design and AM coordinated and helped to draft the manuscript. All authors read and approved the final manuscript.
